# Supplementary material for: Patterns of Intron Gain and Loss in Fungi
Source: PLoS Biol. 2004 Nov 30;2(12):e422. doi: 10.1371/journal.pbio.0020422 (PMC532390; doi:10.1371/journal.pbio.0020422)
Supplement: Table S1 — Also available at http://genes.mit.edu/NielsenEtAl/. (4.3 MB ZIP). [file pbio.0020422.st001.zip › NielsenEtAl/html/665.html]

AN0673.1.NCU07171.1.MG10690.1.FG04485.1


```
 CLUSTAL W (1.82) Multiple Sequence Alignments - Introns Inserted


Sequence 1: MG10690.1	398 aa
Sequence 2: FG04485.1	390 aa
Sequence 3: NCU07171.1	395 aa
Sequence 4: AN0673.1	430 aa
Alignment Length: 444 aa
Number Identitical Residues: 312 aa
Alignment Score (without introns) 12976


MG10690.1 	MAAP-PPIV1LDGGTGFLKVGYAG------------------------QNFPEHQFPSIV
NCU07171.1	MANTDAPIV1LDGGTGFLKVGYAA------------------------QNFPEFQYPSIV
FG04485.1 	MANP-PPIV1LDGGTGFLKVGYAA------------------------QNFPEHQYPSIV
AN0673.1  	-----MPIV1LDGGTGFLKVGYAAQVRLSSPRVSLLSLGLRNDFTLILQNFPEHQFPSIV
          	      *** *************..   ::.  :  : .  .. :   *****.*:****

MG10690.1 	GRPILRTEEKGDNILGPDGNEIKIKDIMCGDEASAARTMLQVSYP~MENGIVKKWDDMQH
NCU07171.1	GRPILRAEEAKSLS---DSSDIVIKDIMCGDEAAAARTMLQISYP~MENGIVKKWDDMQH
FG04485.1 	GRPILRSEEQTD-------SDLVIKDIMCGDEAAAARTMLQISYP~MENGIVKKWDDMEH
AN0673.1  	GRPILRTEEQAG--------DIVVKDIMCGDEAAAARSMLQISYP0MENGIVKKWDDMQH
          	******:**  .        :: :*********:***:***:*** ************:*

MG10690.1 	LWDHTFYEKLKVDPAGRKILLTEPPMNPVKNREQMCEVMFERYNFGGVYVAIQAVLALYA
NCU07171.1	LWDYTFHEKMKVDTRGRKILLTEPPLNPLKNREQMTEVMFERYDFGGVYVAIQAVLALYA
FG04485.1 	LWDYTFYEKLKVDPQGQKILLTEPPMNPLKNREKMCEVMFDRYGFGGVYVAIQAVLALYA
AN0673.1  	LWNYTFYDKMKIDPTGRKILLTEPPMNPLKNREQMAEIMLEHYNFGGVYVAIQAVLALYA
          	**::**::*:*:*. *:********:**:****:* *:*:::*.****************

MG10690.1 	QG1LSSGVVVDSGDGVTHIVPVYESVVLNHLTRRLDVAGRDVTRNLIALLLRRGYALNRT
NCU07171.1	QG1LSSGVVVDSGDGVTHIVPVYESVVLNHLTRRLDVAGRDVTRNLIALLLRRGYALNRT
FG04485.1 	QG1LSSGVVVDSGDGVTHIVPVYESVVLNHLTKRLDVAGRDVTRNLIKLLLRRGYALNRT
AN0673.1  	QG1LSSGVVVDSGDGVTHIIPVYESTVLNHHIRRLDVAGRDVTRNLIALLLRRGYALNRT
          	** ****************:*****.****  :************** ************

MG10690.1 	ADFETVRQIKEKLCYVSYDLELDKRLSEDTTVLVESYTLPDGRVIRVGSERFEAPECLFQ
NCU07171.1	ADFETVRQIKEKLCYVSYDLELDKRLSEDTTVLVESYTLPDGRVIRVGSERFEAPECLFQ
FG04485.1 	ADFETVRQIKEKLCYVSYDLELDKRLSEDTTVLVEDYTLPDGRVIRVGSERFEAPECLFQ
AN0673.1  	ADFETVRQMKEKLAYVSCDLELDRKLSEETTVLVESYTLPDGRVIRVGSERFEAPECLFQ
          	********:****.*** *****::***:******.************************

MG10690.1 	PHLVDCEQKGMGEFLFDTIQAADVDVRSSLFKAIVLSGGSSMYPGLPSRLEKELKQLWLT
NCU07171.1	PHLVDCDQPGIAEFLFNTIQAADVDVRSSLFKAIVLSGGSSMYPGLPSRLEKELKQLWLT
FG04485.1 	PHLVDSESPGLGEFLFNTIQSADVDIRSSLFKAIVLSGGSSMYPGLPSRLEKELKQLWLT
AN0673.1  	PHLVDVDQPGMAELLFNTIQGTDVDVRSSLYKAIVLSGGSSMYPGLPTRLEKELKQLWLT
          	***** :. *:.*:**:***.:***:****:****************:************

MG10690.1 	KVLGGNPERLS~KFKVRIEDPPRRRHMVFLGGAVLANIM---------------------
NCU07171.1	RVLGGNPERLS~KFKVRIEDPPRRRHMVFLGGAVLANIM---------------------
FG04485.1 	RALQGNPERLS~KFKVRIEDPPRRRHMVFLGGAVLANIM---------------------
AN0673.1  	RVLKGDPERLS0KFKVRIEDPPRRRHMVFLGGAVLANLVRSWFPFYCRAHPFTYRIMKQI
          	:.* *:***** *************************:: :  .  . : . :    .. 

MG10690.1 	ADKESMWITKQEWEEQGSRVLEKLGPRS
NCU07171.1	ADKESMWITKQEWEEQGARVLEKLGPR-
FG04485.1 	ADKESMWVTKAEWEEEGTRVLEKLGPR-
AN0673.1  	ADKEDMWVTKQEWEEQGARALAKLGPR-
          	****.**:** ****:*:*.* *****
```
